# Disparities in health and climate change research funding: The funders and the funded

**DOI:** 10.1016/j.joclim.2025.100633

**Published:** 2026-04-04

**Authors:** Jake T.W. Williams, Philomena Colagiuri, Paul J. Beggs, Ying Zhang

**Affiliations:** a University of Sydney School of Public Health, Camperdown, New South Wales, Australia; b School of Natural Sciences, Faculty of Science and Engineering, Macquarie University, Sydney, New South Wales, Australia

**Keywords:** Health and climate change, Grants, Funding, Equity, Research

## Abstract

**Introduction:**

Climate change has significant impacts on health. This study examines global funding for health and climate change research.

**Materials:**

The Dimensions database was used to identify funding awarded between 1990 and 2023 through lower and upper search strategies designed to encompass both focused and more inclusive search approaches.

**Results:**

A total of 1,819 grants were identified in the lower search and 3,326 in the upper search, with total funding ranging from USD1.6–2.6 billion, the majority awarded since 2010. Most grants were issued by government (88–90 %) and non-profit (10–11 %) organisations, primarily from the United States (37 %) and the United Kingdom (15 %). Most funding went to educational research organisations (75 %) in high-income countries (78 %) and in the same country as the funder. No funding went to primary research organisations in low-income countries, and fewer than 1 % went to lower-middle income countries.

**Conclusion:**

While funding for health and climate change research has increased, inequities persist in the global allocation of this research funding. More support is needed for research in low- and lower-middle-income countries to equitably address the health crisis posed by climate change.

## 1 Introduction

Climate change is having a profound effect on human health. Rising global temperatures, more frequent and extreme weather events, higher air pollution, and shifting patterns of infectious disease all contribute to the growing health burden of climate change [1]. Heatwaves are increasing the incidence of heat-related illness and mortality, especially among vulnerable populations [1]. Intensifying storms, floods, and wildfires are leading to injury, displacement, morbidity, and the disruption of essential health services [1]. At the same time, worsening air pollution contributes to respiratory and cardiovascular disease, while changing ecosystems are altering the geographic distribution of vector-borne diseases [1].

Without urgent action, these impacts are expected to escalate, contributing to an estimated 15 million deaths and up to US dollars (USD)15 trillion in economic damage by 2050 [2]. Climate change and health research focuses on understanding these impacts, exploring mitigation and adaptation responses, and examining the broader political, economic, and social context in which these are occurring. Despite a growing number of scientific articles, grants, and funding for research in this field [1,3], climate change and health research remains underfunded relative to the scale of the problem [[3], [4], [5]].

Funding is essential for almost all research to be conducted. Research funding arrangements are complex, and the structure of these arrangements may influence research priorities and outputs [6]. Funding can come from various sources, such as from government bodies or non-profit private organisations, and from funders in the same country as the research organisation, or elsewhere. The relative location of funding and research organisations may also influence the direction of scientific inquiry. This has the potential to exacerbate or alleviate global inequities in addressing the health impacts of climate change [7]. For instance, when research in low- and middle-income countries (LMICs) is primarily funded by institutions in high-income countries (HICs), research agendas may reflect the priorities and perspectives of funders rather than those of LMICs. This could lead to the underrepresentation of region-specific health and climate change research.

A recent global study examined the number and value of grants awarded for climate change and health research between 2000 and 2022 [3]. However, it did not assess the types of funding agencies, their countries of origin, or the field of research, highlighting a gap in understanding how different funding sources and geographic factors influence research support in this field. To date, no work has attempted to characterise the funding agencies and research organisations involved in climate change and health research. This study aims to address this gap by identifying and describing patterns in the funding sources and funded research organisations contributing to climate change and health research.

## 2 Materials and methods

### 2.1 Data

This study uses the Dimensions "Grants" and "Organisations" databases to identify grants awarded for climate change and health research (Digital Science & Research Solutions Inc: London, United Kingdom). Dimensions is the world’s largest research funding database and contains information about more than 7 million grants worth more than USD2 trillion. These data are sourced from more than 700 funding organisations either directly or from public sources. Dimensions was chosen for its global coverage. While national or regional databases may offer more detailed local information, such as research portfolio online reporting tools expenditures and results (RePORTER) for grants from the National Institutes of Health in the United States or community research and development information service (CORDIS) for grants from the European Union Research and Development Projects, they lack the international scope of Dimensions. The Web of Science Grants Index has a similar database, but this includes fewer indexed grants. Dimensions was therefore the most suitable choice for analysing global climate change and health research funding available for this study.

### 2.2 Search strategy

We searched the title and abstract of grants that started between 1990 and 2023 in the Dimensions database. The search was performed by two authors (JW and PC) on 25 November 2024. Previous studies have highlighted challenges identifying climate change and health research [3,8]. To address these we used "upper" and "lower" search strategies, consistent with previous work [8]. The lower strategy was designed to be conservative and aimed to find only grants that were clearly related to both climate change and human health. It contained two search terms: “climate change” and “human health”. The upper strategy was designed to be more inclusive of climate change and human health related grants and used sets of terms related to climate change and human health. Climate change-related terms included *“climate change,” “global warming,” “greenhouse gas,”* and *“climate policy.”* Health-related terms focused on outcomes for humans and included proximity-based phrases such as *“human health,” “human disease,” “human mortality,” “human illness,”* and *“human wellbeing.”* (see Appendix A). Search terms were identified from previous work [3,8,9]. The upper search strategy required the inclusion of at least one health term and one climate change term, and proximity searching was applied to the health terms. This was done to capture grammatical variants (e.g., human health and health of human populations). Proximity searching was not used in the lower search strategy. The identified grants were not screened for eligibility, and all results of the upper and lower search were included in this study, consistent with similar high-level database studies.

### 2.3 Grants

Data about each grant that was used in this analysis included funding amount in USD (converted by Dimensions from the original currencies to USD based on the exchange rate at the time of the grant); start and end date of grant; name and location of funder; name and location of funded research organisation; and field of research. Funding amount in USD was inflated from the grant’s start year to 2023 using average All Urban Consumers Consumer Price Index (CPI) from the US Bureau of Labor Statistics [10]. Countries were classified according to the appropriate World Bank income groups for the 2024–25 financial year (low-income, lower-middle-income, upper-middle-income, or high-income economy) [11]. Funded research organisations were classified into one of the following categories using the Dimensions "Organisations" database: healthcare, education, archive, facility, non-profit, company, government, other. Definitions of these categories are available in Appendix B. Where more than one research organisation was recorded, the first listed organisation was assumed to be the primary research organisation and all others were classified as secondary research organisations. The field of research was classified according to the Australian and New Zealand Standard Research Classification (ANZSRC) 2020. We described the ANZSRC division of research of each grant.

### 2.4 Statistical analysis

Descriptive statistics were reported for all variables included in this study. Missing values were excluded from the calculation of summary statistics. For each search strategy, the number of grants and amount awarded was reported by year, funder, and primary research organisation. The secondary research organisations were described. The funding organisations that awarded the most grants were identified. Data were organised and analysed using R version 4.1.4 (R Foundation for Statistical Computing: Vienna, Austria) and R Studio version 2024.09.0 + 375 (Posit: Boston, United States).

## 3 Results

A total of 1,819 grants were identified in the lower search and 3,326 grants were identified in the upper search (Table 1). The total amount awarded over the study period was USD1.6 billion to USD2.6 billion (lower to upper search) and the median grant amount was USD232,000 to USD259,000 (noting that grant amount was missing for 23 % to 26 % of grants). The most common field of research categories that Dimensions assigned to the grants were the environmental (877[48 %]−1509[45 %]) and earth (547[30 %]−750[23 %]) sciences.

**Table 1:** Descriptive statistics of climate change and human health grants included in this study.

	Lower search (*n* = 1819)	Upper search (*n* = 3326)
Start year, n ( %)		
1990–1994	2 (0)	3 (0)
1995–1999	14 (1)	20 (1)
2000–2004	39 (2)	67 (2)
2005–2009	201 (11)	331 (10)
2010–2014	500 (28)	835 (25)
2015–2019	469 (26)	906 (27)
2020–2023	594 (33)	1,164 (35)
Amount in 2023 USD		
Median	231,812	259,183
Q1, Q3	102,861,673,639	96,162,708,768
Total	1,606,124,177	2,589,247,962
Missing, n ( %)	423 (23)	877 (26)
ANZSRC 2020 Division, n ( %)^a^		
Environmental Sciences	877 (48)	1,509 (45)
Earth Sciences	547 (30)	750 (23)
Biological Sciences	365 (20)	776 (23)
Agricultural, Veterinary and Food Sciences	217 (12)	502 (15)
Health Sciences	175 (10)	270 (8)
Human Society	126 (7)	240 (7)
Biomedical and Clinical Sciences	101 (6)	250 (8)
Other	430 (24)	967 (29)

ANZSRC: Australian and New Zealand Standard Research Classification, USD: United States Dollar.

a Proportions do not add to 100 % because a grant can belong to more than one category.

**Fig. 1:**
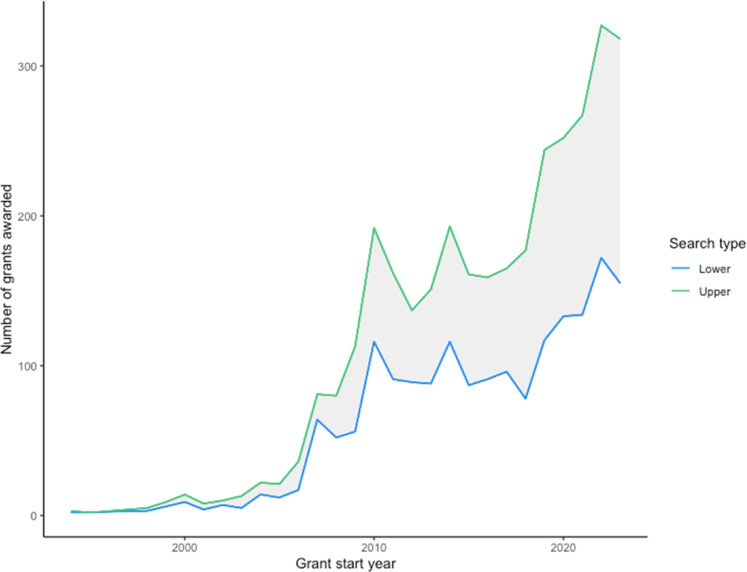
Total annual number of grants awarded globally for climate change and human health research between 1990 and 2023. The green line is the result from the upper search strategy and the blue line is the result from the lower search strategy.

**Fig. 2:**
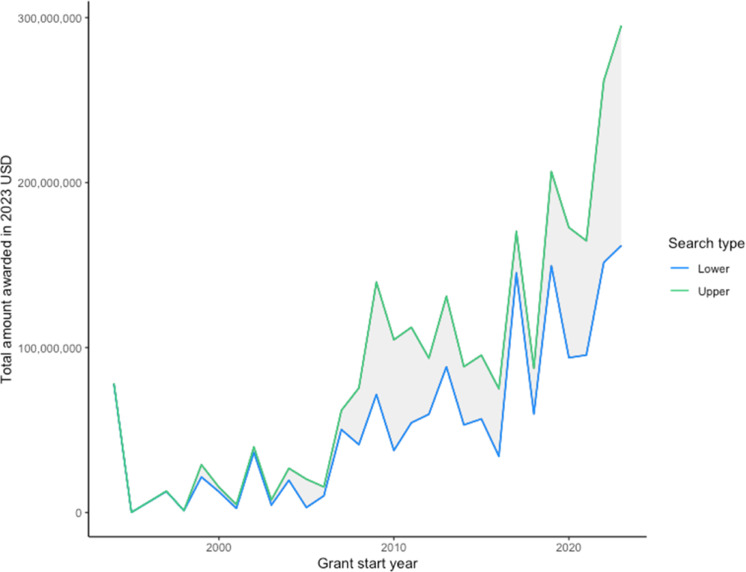
Total annual grant funding (2023 USD) awarded globally for climate change and human health research between 1990 and 2023. The green line is the result from the upper search strategy and the blue line is the result from the lower search strategy.

The number of grants and amount of funds awarded over time has increased (Fig. 1, Fig. 2). There were few grants from 1990 to 2006. A period of rapid growth in grants occurred over the period 2007–2010. Grants then plateaued until 2017–18, after which there was a second surge in funding.

Most grants (shown for grants identified through lower to upper search) were funded by governments (1,628[90 %]−2,941[88 %]) and non-profit organisations (182[10 %]−369[11 %]). Funders were mainly based in the United States (670[37 %]−1,125[34 %]) and the United Kingdom (277[15 %]−494[15 %]). All funding organisations were based in high-income (1,564[86 %]−2,847[86 %]) or upper middle-income (255[14 %]−479[14 %]) countries, with no funder organisations based in lower-middle income (0 %) or low-income countries (0 %). Details of funding organisations are reported in Table 2.

**Table 2:** Descriptive statistics of climate change and human health grants funders included in this study.

	Lower search (*n* = 1819)	Upper search (*n* = 3326)
Funder organisation type, n ( %)		
Government	1,628 (90)	2,941 (88)
Non-profit	182 (10)	369 (11)
Other^a^	9 (1)	16 (1)
Funder country, n ( %)		
United States	670 (37)	1,125 (34)
United Kingdom	277 (15)	494 (15)
Canada	173 (10)	341 (10)
Brazil	126 (7)	277 (8)
Belgium^b^	97 (5)	204 (6)
China	97 (5)	140 (4)
Other^c^	379 (21)	745 (22)
Funder country income group, n ( %)		
High income	1,564 (86)	2,847 (86)
Upper middle income	255 (14)	479 (14)
Lower middle income	0	0
Low income	0	0

a Includes healthcare, education, archive, facility, company, and other categories from Dimensions database.

b Belgium is the location of the European Union’s primary institutions and grants awarded by these funders are attributable to Belgium in the Dimensions database and this analysis.

c Full details are provided in the Appendix.

The top five government and non-profit funding organisations by number of grants are listed in Table 3. Globally, the United States Environmental Protection Agency and United Kingdom Natural Environment Research Council were the most prolific government funders and Brazil’s Sao Paulo Research Foundation was the most prolific non-profit funder. The largest grant identified in this study was awarded by the US National Institute of Environmental Health Sciences to the University of Arizona for the Southwest Environmental Health Sciences Center.

**Table 3:** The leading government and non-profit funders of climate change and human health research from 1990 to 2023 in terms of the number of grants awarded (ordered according to the number of grants found in the lower search strategy).

Government			Non-profit		
Funder	Country	Number of grants	Funder	Country	Number of grants
Environmental Protection Agency	United States	Upper *n* = 183 Lower *n* = 151	São Paulo Research Foundation	Brazil	Upper *n* = 81 Lower *n* = 43
Natural Environment Research Council	United Kingdom	Upper *n* = 219 Lower *n* = 143	Fundação para a Ciência e Tecnologia	Portugal	Upper *n* = 64 Lower *n* = 33
Natural Sciences and Engineering Research Council	Canada	Upper *n* = 263 Lower *n* = 134	Wellcome Trust	United Kingdom	Upper *n* = 34 Lower *n* = 25
National Institute for Food and Agriculture	United States	Upper *n* = 206 Lower *n* = 112	Deutsche Forschungsgemeinschaft	Germany	Upper *n* = 39 Lower *n* = 12
Directorate for Geosciences	United States	Upper *n* = 158 Lower *n* = 111	Russian Science Foundation	Russia	Upper *n* = 22 Lower *n* = 12

**Fig. 3:**
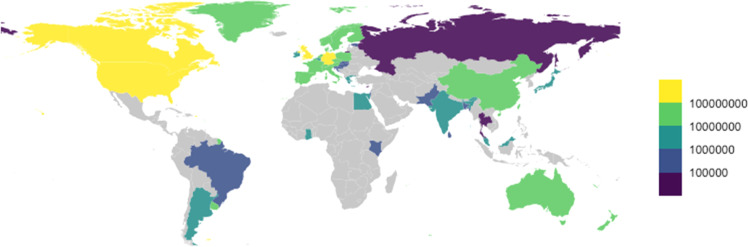
Total funding (in 2023 USD) awarded for climate change and human health research between 1990 and 2023, by the country of primary research organisation based on the upper search strategy. Countries are grouped on a log10 scale. Grey-shaded countries indicate where no grant was identified with a primary research organisation in that country.

Grants were mostly awarded to educational organisations (1,365[75 %]−2,479[75 %]) and organisations based in the United States (640[35 %]−1,076[32 %]) and United Kingdom (252[14 %]−469[14 %]) (Table 4). Total funding awarded to research organisations in the United States was USD963 million, more than for any other country (Fig. 3). Primary research organisations were mostly based in high-income countries (1,425[78 %]−2,591[78 %]) and upper middle-income countries (242[13 %]−449[14 %]), with fewer than 1 % of grants (6–10) awarded to organisations in lower middle-income counties. No grant identified in this study was awarded primarily to a research organisation based in a low-income country (0; 0 %). Most grants were awarded to primary research organisations based in the same country as the funder (1,581[87 %]−2,881[87 %]). Secondary research organisations were identified for 15 % to 16 % of grants, and 90 % of these (1,220–2,029) were based in high income countries with the remainder from upper-middle income countries (95[7 %]−148[7 %]) and lower-middle income countries (44[3 %]−80[4 %]), and just a few based in low-income countries (3[0 %]−13[1 %]).

**Table 4:** Descriptive statistics of climate change and human health grant research organisations included in this study.

	Lower search (*n* = 1819)	Upper search (*n* = 3326)
Primary research organisation type, n ( %)		
Education	1,365 (75)	2,479 (75)
Facility	105 (6)	192 (6)
Government	68 (4)	132 (4)
Non-profit	81 (5)	139 (4)
Other	54 (3)	108 (3)
Missing	146 (8)	276 (8)
Primary research organisation country, n ( %)		
United States	640 (35)	1,076 (32)
United Kingdom	252 (14)	469 (14)
Canada	172 (10)	340 (10)
Brazil	125 (7)	275 (8)
China	97 (5)	141 (4)
Other^b^	387 (21)	749 (23)
Missing	146 (8)	276 (8)
Primary research organisation country income group, n ( %)		
High income	1,425 (78)	2,591 (78)
Upper middle income	242 (13)	449 (14)
Lower middle income	6 (0)	10 (0)
Low income	0	0
Missing	146 (8)	276 (8)
Primary research organisation is in the same country as the funder organisation		
Yes	1,581 (87)	2,881 (87)
No	92 (5)	169 (5)
Missing	146 (8)	276 (8)
Number of research organisations listed on grant, n ( %)		
1	1,388 (76)	2,552 (77)
2	117 (6)	203 (6)
3	51 (3)	94 (3)
4	27 (2)	45 (1)
5+	90 (5)	156 (5)
Missing	146 (8)	276 (8)
Total number of secondary research organisations, n	1,362	2,270
Secondary research organisations country, n ( %)^a^		
United Kingdom	377 (28)	605 (27)
United States	113 (8)	146 (6)
Germany	81 (6)	142 (6)
France	67 (5)	130 (6)
Other	724 (53)	1,247 (55)
Secondary research organisations country income group, n ( %)^a^		
High income	1,220 (90)	2,029 (90)
Upper middle income	95 (7)	148 (7)
Lower middle income	44 (3)	80 (4)
Low income	3 (0)	13 (1)

a Data reported as a proportion of the total number of secondary research organisations.

b Full details are provided in the Appendix.

Further details from the analysis are provided in Appendix C.

## 4 Discussion

In this study, we identified grants awarded for climate change and human health research from 1990 to 2023 and described the funding and research organisations involved. Total funding identified ranged from USD1.6 billion (lower search) to USD2.6 billion (upper search). Both the number of grants and the amount of funds awarded annually has increased substantially over time. Most funders were government organisations from HICs, and most of the funded research organisations were educational organisations and those based in HICs, primarily within the same country as the funder. Notably, no grants were led by a primary research organisation in a low-income country (LIC), and less than 1 % were awarded to organisations in lower-middle income countries. Grants were most commonly categorised by the Dimensions database as being in the Environmental and Earth Sciences.

Our findings are consistent with a recent analysis by Sovacool et al., who reported an increase in both the value of grants awarded for climate change and health research and the proportion of these grants within the Dimensions database [3]. However, while Sovacool et al. focused on overall trends in funding, they did not examine *who* is funding this research and *where* the research is being conducted. To our knowledge, our study is the first to systematically investigate the characteristics of both funders and recipient research organisations in the climate change and health research landscape, including their geographic location, income classification, and institutional type. This additional layer of analysis reveals deep structural inequities that have not been previously examined.

The observed rise in research funding is consistent with broader global (non-research) financing and research outputs. A 2023 report observed a rise in funding commitments since 2018, with examples such as the UK’s pledge of approximately USD25 million to support research on climate-resilient health systems [12]. This rise in funding coincides with an increase in the number of research articles in the field, with the *Lancet* Countdown indicator tracking scientific articles on health and climate change reaching its highest recorded level in 2023 [1].

A review of climate change and health literature between 2013 and 2020 found that most of these studies were from HICs and upper middle-income countries, which is consistent with our findings [13]. This is consistent with broader health research funding, with a 2020 analysis by the World Health Organization revealing stark disparities in global health research funding, resource allocation, and capacity between HICs and LMICs [14]. Most global health research funding originates from HICs, which predominantly fund institutions within their own borders. For example, Charani et al. have highlighted that 80 % of USAID contracts go to US firms, and 88 % of Bill and Melinda Gates Foundation (BMGF) grants are held by institutions in the Global North. Major donors such as the US and European country governments, and philanthropies like BMGF, are highly influential in shaping the global health agenda [15]. HICs also continue to dominate research output, producing nearly 80 % of global health publications, despite representing only 16 % of the global population and accounting for just 10 % of the global disease burden [16].

There are several likely reasons for this distribution of research funding. There are significant differences in research capacity, as HICs have approximately 56 times more health researchers per million people and greater access to higher education than LICs [14]. Researchers in HICs are generally better equipped to navigate the academic research system, including grant writing and publishing, which contributes to the underrepresentation of LMIC researchers on funding applications [15]. LMIC partners are also often underrepresented on funding panels, limiting their influence on funding decisions [17]. The dominance of English in academic publishing may further disadvantage non-English speaking LMIC researchers, creating additional barriers [18]. Finally, databases like Dimensions may underrepresent LMIC-based funders and institutions due to inconsistent reporting or a lack of visibility, reinforcing a distorted view of the global funding landscape.

Despite the growth in funding for climate change and health research, overall research funding levels [4] and its allocation [8] may still be insufficient to meet international climate commitments and address the scale of the health risks posed by climate change. HICs, while historically responsible for the bulk of greenhouse gas emissions, are unlikely to experience the most severe health impacts of climate change. Instead, LMICs are expected to bear the greatest burden [19], yet they receive only a fraction of the funding. The cost of funding for climate change and health research in these settings is far exceeded by the potential cost of inaction [2].

Since HIC governments are funding most research in this field, there is a risk that issues aligned with their own political and economic interests may be prioritised, while the urgent needs of other regions remain overlooked. The lack of direct funding to many LMICs contributes to a knowledge deficit in these regions, creating gaps in global understanding, particularly as the impacts of climate change—such as migration and the spread of vector-borne diseases—are not confined to national borders [1]. A recent study found that funding for climate change research in Africa is largely driven by funder organisations from the European Union, the United Kingdom, and the United States [20], potentially shaping research agendas from a Northern perspective. Given that LICs are already bearing the brunt of climate change’s health impacts, it is crucial that funding for research in these areas be guided by local priorities. This approach is essential for addressing global climate change and health challenges more equitably.

Charani et al. proposed opportunities for funders to address inequalities in global health research and we believe these can apply to research in climate change and health. This could include funders committing to allocate funds equitably based on need, funding investigators who may be disadvantaged, and developing criteria of funding eligibility broader than just academic outputs [15].

Despite its strengths, this study has several limitations. While we believe it is the best option available, the Dimensions database does not encompass all grants and organisations that fund research globally. Since government data are often publicly available and systematically reported, government funders are more likely to be indexed in the database and therefore overrepresented in our analysis. Similarly, organisations in HICs and documents originally in English are over-represented in the database. Finally, about a quarter of grants were missing data on the funding amount. This will have resulted in an underestimation of the total amount of funding in this field and other inaccuracies in the results.

While the data on which this analysis is based precede the commencement of the second Trump administration in the US, our results put a number of actions of that administration into context. Over the 34 years of our analysis (1990 and 2023) the US was the leading funder of climate change and health research, funding over a third of grants in this field. This means that the current US administration's termination of climate change and health grants [[21], [22], [23]] will have substantial adverse impacts on progress in this field, resulting in disease and deaths (in the US and globally) that would otherwise have been avoided [1]. The sudden reversal of US policy on climate change and health research funding by the Trump administration in 2025 also means the results of our study cannot be extrapolated beyond 2023.

## 5 Conclusion

Funding for climate change and health research has increased substantially since 1990 and has largely been awarded by public funders to research in HICs. No grants were awarded to primary research organisations in LICs, and less than 1 % were awarded to lower middle-income countries. To equitably address the scale of the health crisis posed by climate change, greater investment is needed in LMICs to build local research capacity. Ensuring more inclusive funding flows is essential for developing context-specific solutions and advancing climate and health equity.

This article is featured in an Editorial in this issue, which includes comment on this work.

## CRediT authorship contribution statement

**Jake T.W. Williams:** Writing – review & editing, Writing – original draft, Methodology, Investigation, Formal analysis, Data curation, Conceptualization. **Philomena Colagiuri:** Writing – review & editing, Writing – original draft, Methodology, Investigation, Data curation, Conceptualization. **Paul J. Beggs:** Writing – review & editing, Supervision, Project administration, Methodology, Funding acquisition, Conceptualization. **Ying Zhang:** Writing – review & editing, Supervision, Project administration, Methodology, Funding acquisition, Conceptualization.

## Declaration of competing interest

The authors declare that they have no known competing financial interests or personal relationships that could have appeared to influence the work reported in this paper.

## Appendix Supplementary materials

Image, application 1

## Footnotes

Supplementary material associated with this article can be found, in the online version, at doi:10.1016/j.joclim.2025.100633.
