# Supplementary material for: Disparities in health and climate change research funding: The funders and the funded
Source: J Clim Chang Health. 2026 Apr 4;27:100633. doi: 10.1016/j.joclim.2025.100633 (PMC13090324; doi:10.1016/j.joclim.2025.100633)
Supplement: Supplementary file 1 [file mmc1.pdf]

Appendices

Appendix A - Search strategy and terms used to find climate change and human health grants in the Dimensions database	Page 2
Appendix B – Funded organisation categories taken from Dimensions	Page 3
Appendix C – detailed results tables	Pages 4-8

Appendix A - Search strategy and terms used to find climate change and human health grants in the Dimensions database

Strategy Type	Title and Abstract Search
Lower search	“climate change” AND “human health”
Upper search	(“climate change” OR “global warming” OR “greenhouse effect” OR “greenhouse gas” OR GHG OR “CO2 emissions” OR “climate policy”)<br><br>AND<br><br>("human health"~5 OR "human disease*"~5 OR "human death*"~5 OR "human morbidit*"~5 OR "human mortalit*"~5 OR "human ill"~5 OR "human illness*"~5 OR "human infect*"~5 OR "human medical*"~5 OR "human well?being"~5)

Note: ~n is a proximity search term which looks for results where the words within the health terms are a maximum distance of n number of words apart (in any order). The ? search term is a single character wildcard.

Appendix B – Funded organisation categories taken from Dimensions

Type	Description	Implementation	Example
Healthcare	A health-related facility where primarily patients are treated. Includes hospitals, medical centers, health centers, treatment centers. Also includes trusts and healthcare systems.	An organised provision of healthcare as the primary function will get Healthcare assigned.	A private hospital with educational responsibilities will be categorised as Healthcare.
Education	An educational institution where research takes place. Can grant degrees and may have faculties, departments, and schools.	Is used for any public or private organisation where the primary task is education.	A public university with a medical school is categorised as Education.
Archive	Repository of documents, artefacts, or specimens. Includes libraries and museums that are not part of a university.	Primary function is a research archive.	A place where information and records are stored that allows access to the collection for research purposes is categorised as Archive.
Facility	A building or facility dedicated to research within a specific area. Usually contains specialised equipment. Includes specialist research institutes as well as laboratories and large infrastructures such as astronomical observatories and particle accelerators.	Primary function is research facility.	An astronomical observatory that provides a library service is categorised as Facility.
Non-profit	An organisation that uses its surplus revenue to achieve its goals. Includes charities and other non-government research funding bodies.	A non-business entity operated for a collective, public or social benefit.	A charitable organisation that carries out clinical research is categorised as Non-profit.
Company	A legal entity with the aim of engaging in business and gaining profit.	Primary function is profit-making business.	An organisation that sells healthcare products and conducts research is categorised as Company.
Government	An organisation operated mainly by the government of one or multiple countries / territories.	Any designation that is used by a government such as a ministry, department, secretariat, office, directorate or federal agency.	A government agency for healthcare is categorised as Government.
Other	Used in cases where none of the other aforementioned types are suitable.	Anything not belonging to any of the other categories, or where the primary function is unclear.	A university press would fall into this category.

Appendix C – detailed results tables

Table C1. Total number of grants and funds awarded (2023 USD reported to 0 decimal places) by the start year of the grant, for years between 1990 and 2023.

Start year of grant	Lower search		Upper search	
	Number	Amount awarded (2023 USD)	Number	Amount awarded (2023 USD)
1990	0	0	0	0
1991	0	0	0	0
1992	0	0	0	0
1993	0	0	0	0
1994	2	77895664	3	78297982
1995	2	119961	2	119961
1996	0	0	0	0
1997	3	12810405	4	13000250
1998	3	1167242	5	1167242
1999	6	21520466	9	28990413
2000	9	12619110	14	15408226
2001	4	2518719	8	4880368
2002	7	36415058	10	39754871
2003	5	4503885	13	7723717
2004	14	19566075	22	26840999
2005	12	3033431	21	20270482
2006	17	10155403	36	15550346
2007	64	50277601	81	61938179
2008	52	41149038	80	75541822
2009	56	71553502	113	139768275
2010	116	37579643	192	104743209
2011	91	54431764	162	112298309
2012	89	59557485	137	93620942
2013	88	88306178	151	131097127
2014	116	53111635	193	88414270
2015	87	56732529	161	95413575
2016	91	34039554	159	75073627
2017	96	145219876	165	170443977
2018	78	59677145	177	87494466
2019	117	149489936	244	206682574
2020	133	93846961	252	172814990
2021	134	95430592	267	164725895
2022	172	151580474	327	261959321
2023	155	161814846	318	295212547

Table C2. Total number of grants and funds awarded (2023 USD reported to 0 decimal places) by the funder organisation country.

Funder organisation country	Lower search		Upper search	
	Number	Amount awarded (2023 USD)	Number	Amount awarded (2023 USD)
Australia	24	23685819	41	33013448
Austria	9	2573956	25	5986010
Bahamas	1	2204902	1	2204902
Belgium	97	261173795	204	516518335
Brazil	126	0	277	0
Canada	173	69139577	341	102512901
Chile	2	0	2	0
China	97	11333856	140	14952637
Croatia	2	358956	8	1128158
Czechia	9	49874088	10	49991227
Denmark	2	629024	2	629024
Estonia	2	240238	3	375046
Finland	30	12049399	50	21611779
France	15	11838684	31	21423208
Germany	16	279102	45	436242
Hungary	2	125681	3	150864
Israel	2	386053	3	600086
Italy	3	0	7	0
Japan	6	4565127	29	7444266
Luxembourg	1	0	1	0
Malaysia	7	0	9	0
Netherlands	6	0	9	186276
New Zealand	4	64428056	5	64777111
Norway	27	29869131	44	36024718
Poland	22	18117143	51	30336491
Portugal	33	54936399	64	68764882
Qatar	4	0	6	0
Russia	46	0	87	0
Slovakia	3	358752	6	740435
Slovenia	15	0	21	0
South Africa	25	0	53	0
Spain	1	92886	1	92886
Sweden	25	8995959	59	26770853
Switzerland	35	44922070	69	76151386
United Kingdom	277	325688043	494	506882031
United States	670	608257485	1125	999542760

Table C3. Total number of grants and funds awarded (2023 USD reported to 0 decimal places) by the funder organisation type.

Funder organisation type	Lower search		Upper search	
	Number	Amount awarded (2023 USD)	Number	Amount awarded (2023 USD)
Archive	1	186993	1	186993
Education	1	4414760	1	4414760
Facility	4	4151209	9	17277965
Government	1628	1429358028	2941	2348059125
Healthcare	3	1652344	5	2065137
Non-profit	182	166360844	369	217243982

Table C4. Total number of grants and funds awarded (2023 USD reported to 0 decimal places) by the primary research organisation country.

Primary research organisation country	Lower search		Upper search	
	Number	Amount awarded (2023 USD)	Number	Amount awarded (2023 USD)
Argentina	1	2173180	1	2173180
Australia	25	37587111	40	46914741
Austria	8	2573956	22	15966705
Bangladesh	0	0	1	738289
Belgium	29	201877	59	6473293
Bermuda	3	1056065	3	1056065
Brazil	125	306183	275	306183
Canada	172	69115102	340	102231063
Chile	2	0	2	0
China	97	11333856	141	15120188
Croatia	2	358956	5	955903
Cyprus	1	87662	1	87662
Czechia	8	43839256	11	46961460
Denmark	8	48108897	16	67260956
Egypt	0	0	1	3407207
Estonia	3	671754	4	806563
Finland	25	12383838	46	41861725
France	20	26951384	44	86025681
Germany	26	89996449	57	102354069
Ghana	1	3213021	1	3213021
Greece	2	7600186	3	7812995
Hungary	2	125681	3	150864
India	2	1646621	3	1646621
Iran	1	0	1	0
Ireland	1	238939	3	3065381
Israel	1	239516	2	453548
Italy	7	49118295	13	61532973
Jamaica	1	0	1	0
Japan	6	1612433	29	4491572
Kenya	2	440703	2	440703
Luxembourg	1	0	1	0
Malaysia	8	2483783	10	2483783
Netherlands	9	10119239	16	33941363
New Zealand	5	66843745	6	67192800
Norway	20	29250286	35	58491095
Pakistan	0	0	1	230863
Poland	23	18205845	51	30389231
Portugal	33	71072704	64	90466427
Qatar	4	0	6	0
Russia	13	40229	22	40229
Singapore	0	0	1	119165
Slovakia	3	358752	6	740435
Slovenia	16	414222	22	414222
South Africa	8	0	19	0
Spain	5	11800533	9	32792127
Sri Lanka	1	257264	1	257264
Sweden	26	9230619	61	27005513
Switzerland	23	14412971	42	23943705
Thailand	1	31066	1	31066
United Kingdom	252	290123344	469	499531599
United States	640	578650560	1076	963309436
Uruguay	1	18904482	1	18904482
Missing	146	72943614	276	115454544

Table C4. Total number of grants and funds awarded (2023 USD reported to 0 decimal places) by the primary research organisation type.

Primary research organisation type	Lower search		Upper search	
	Number	Amount awarded (2023 USD)	Number	Amount awarded (2023 USD)
Archive	8	3218081	19	19760869
Company	36	51668771	72	104544465
Education	1365	1127026647	2479	1799479998
Facility	105	149550009	192	226236316
Government	68	91529550	132	143237643
Healthcare	9	20124328	14	27962613
Non-profit	81	89994783	139	151899732
Other	1	68395	3	671783
Missing	146	72943614	276	115454544
